# The role of p38 MAPK in acteoside-mediated gastric ulcer protection

**DOI:** 10.7150/ijms.128817

**Published:** 2026-02-04

**Authors:** Yng-Tay Chen, Yun-Ju Lin, Cheng-Hsiang Tsai, Yu-Ching Lu

**Affiliations:** 1 Graduate Institute of Food Safety, College of Agriculture and Natural Resources, National Chung Hsing University, Taichung City 402202, Taiwan.; 2 Department of Food Science and Biotechnology, College of Agriculture and Natural Resources, National Chung Hsing University, Taichung City 402202, Taiwan.

**Keywords:** *Anisomeles indica*, acteoside, pyloric ligation, gastric ulcer, p38 MAPK

## Abstract

Stomach ulcers are a significant health concern, with epidemiological studies indicating multiple forms and causes. Current treatments often lead to various side effects and may require additional medical resources. Therefore, there is an urgent need to identify safer natural extracts with fewer side effects. In this study, we established two models: an *in vivo* model, where gastric ulcer was induced through pyloric ligation in Wistar rats, and an *in vitro* model, where indomethacin treatment in RGM1 gastric mucosal cells was used to evaluate the gastroprotective effects of *Anisomeles indica* (L.) Kuntze HP813 powder (AIHP) *in vivo*, and its major functional component, acteoside, protective effects mechanisms *in vitro*. The preventive gastroprotective effect of AIHP was assessed in Wistar rats with pyloric ligation-induced ulcers. We evaluated the ulcer index, gastric acidity, and the immunohistochemical expression of anti-inflammatory markers in gastric tissues. We evaluated the protective effects and the underlying mechanism of acteoside in indomethacin-treated RGM1 cells, utilizing transient transfection with shRNA targeting p38 mitogen-activated protein kinase (p38 MAPK) to specifically examine its role. Results showed AIHP significantly reduced ulcer index and downregulated TNF-α, IL-1β, NF-κB, and p38 MAPK expression in gastric tissue. Acteoside significantly increased the cell viability and down-regulated TNF-α, IL-1β, IL-6, NF-κB, and p38 MAPK expression of RGM1 cells after indomethacin treatment. Following p38 MAPK knockdown via shRNA, acteoside reduced the indomethacin-induced expression of p38 MAPK and IL-6 in RGM1 cells. These effects are mediated through the suppression of inflammatory mediators via the p38 MAPK pathway. Our findings support acteoside's potential as a pharmacological agent for the management of gastric ulcers.

## Introduction

Gastric ulcer remains a prevalent gastrointestinal disorder, affecting millions of individuals worldwide and imposing a significant burden even on otherwise healthy populations 1,2. Individuals with gastric ulcers are at a higher risk of developing gastric adenocarcinoma 3. Gastric ulcers can be induced by alcoholism, smoking, stress, excessive gastric acid secretion, and adverse drug reactions 4,5,6. Increasing evidence implicates inflammation as a central mechanism in the pathogenesis of gastric ulcers 7.

Excess gastric acid secretion is frequently associated with infection by *Helicobacter pylori* (*H. pylori*), which can survive in acidic environments and exacerbate inflammatory responses in the stomach by secreting toxins and proinflammatory factors 8,9. Notably, *H. pylori* infection is closely linked to the onset of gastric ulcers and chronic gastritis 10. Excessive gastric acid can directly damage the gastric mucosa-a protective layer that lines the stomach and consists of mucus and bicarbonate ions, which help resist the erosive effects of gastric acid 11,12. This protective mechanism is compromised in individuals with excessive gastric acid secretion, resulting in erosion and damage to the gastric mucosa and ultimately inducing the development of ulcers 13. A highly acidic stomach environment can also lead to oxidative stress 14. Gastric mucosal damage results in the production of free radicals and other oxidative molecules, which further impair cellular structures and trigger inflammatory responses 15. Oxidative stress activates multiple signaling pathways, including the nuclear factor-κB (NF-κB) pathway, a major regulator of proinflammatory cytokines such as tumor necrosis factor-α (TNF-α), interleukin-1β (IL-1β), and IL-6 16. The acidic environment and oxidative stress also activate immune cells in the gastric mucosa, particularly macrophages and neutrophils 17. p38 mitogen-activated protein kinases (p38 MAPK), key members of the mitogen-activated protein kinase (MAPK) family, play central roles in cellular responses to stress, inflammation, and immune regulation 18. These kinases are activated by various stimuli, including tumor necrosis factor-α (TNF-α) and interleukin-1β (IL-1β) 19.

*Anisomeles indica* (*A. indica*) (L.) Kuntze, a plant in the family Lamiaceae, is widely used as a traditional herbal medicine in Asia for treating gastrointestinal diseases, including gastric ulcers and H. pylori-related inflammation 20,21,22,23,24. The primary active ingredients in *A. indica* extracts include apigenin, ovatodiolide, β-sitosterol, and acteoside 25,26. Pharmacologically, these compounds can reduce inflammation, inhibit tumor cell growth, suppress *H. pylori* infection, and treat various gastrointestinal, liver, and inflammatory skin diseases 27,28. Previous studies have identified acteoside as an active chemical constituent of *A. indica* extracts 23. Acteoside has been shown to ameliorate ethanol-induced gastric ulcers by modulating the IkB-a and NF-κB pathways 24. However, the efficacy and the precise anti-inflammatory mechanism of A. indica in treating gastric ulcers primarily driven by excessive gastric acid secretion remain to be fully elucidated. In the Shay ulcer rat model, pyloric ligation induces a series of pathophysiological changes, such as gastric content retention, excessive gastric acid secretion, and gastric mucosal damage, thereby triggering local inflammatory responses 1,2,29. These inflammatory responses are closely related to immune cell activation, oxidative stress, proinflammatory cytokine release, and changes in microvascular permeability 30. The interplay among these mechanisms may ultimately lead to gastric diseases such as ulcers and bleeding.

In this study, we explored the ulcer-protective potential of *A. indica* HP813 powder (AIHP) following pyloric ligation in a rat model and investigated whether acteoside regulates inflammatory pathways in RGM1 cells to evaluate AIHP's potential as a gastroprotective drug.

## Material and Methods

### Sample

AIHP was provided by SYI Biotechnology (Taichung, Taiwan). It was formulated with corn-derived soluble fiber, xylo-oligosaccharides, and powder extracted from *A. indica*. The key functional ingredients were xylo-oligosaccharides and acteoside. The preparation exhibited good water solubility, with acteoside quantified at 55 μg per 2,000 mg of AIHP. Acteoside used for cell treatment was also provided by SYI Biotechnology (Taichung, Taiwan).

### Gastroprotection animals

**Figure 1:**
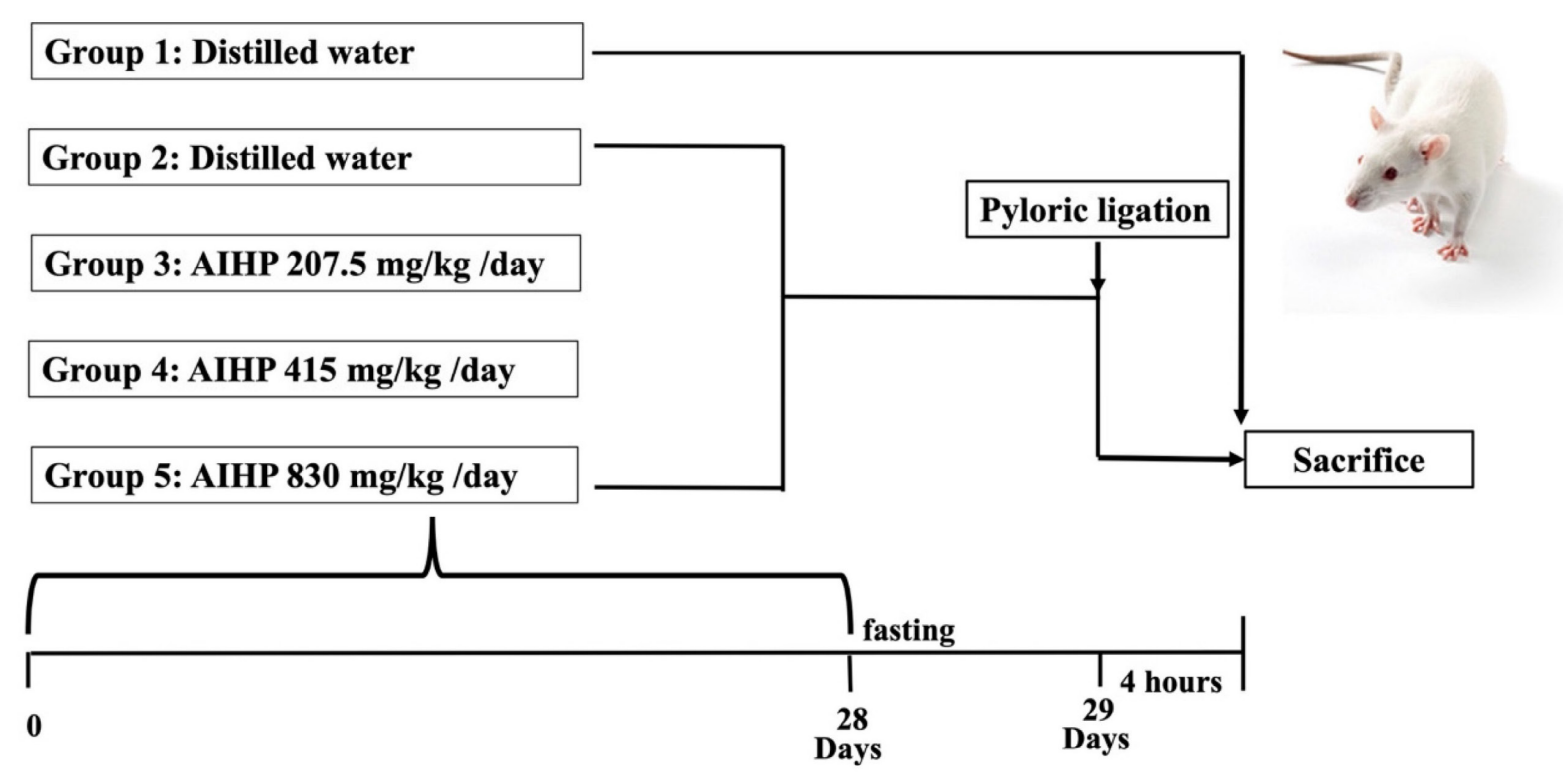
** Animal experimental procedure for the evaluation of gastrointestinal functional improvement due to AIHP in the pyloric ligation-induced gastric ulcer rat model**.

Male Wistar rats (5 weeks old) were obtained from BioLASCO Taiwan (Yilan, Taiwan). The gavage dose was 10 mL/kg body weight and was adjusted weekly. The recommended daily intake of AIHP for an adult weighing about 60 kg is 4,000 mg, equivalent to 67 mg/kg/day. For rats, multiplying the recommended dose by 6.2 (Km factor = 6.2 for rats) yields a dose of 415 mg/kg/day. A dose of 830 mg/kg represents twice the dose, whereas 207.5 mg/kg is half the dose. Details of the experimental groups are shown in Figure 1. All animal experiments were conducted in accordance with the NIH (National Research Council) Guide for the Care and Use of Laboratory Animals, and were approved by the Institutional Animal Care and Use Committee (IACUC) of National Chung Hsing University (Approval No. 113-117). The study design, conduct, and reporting complied with the ARRIVE (Animal Research: Reporting of In Vivo Experiments) guidelines.

### Pyloric ligation-induced gastric ulcer

The rat gastric ulcer model was established using pyloric ligation, as described in a previous study 29. Animals were randomly assigned to experimental groups using a simple randomization method before the initiation of treatment. After 7 days of acclimation, the rats were divided into five groups (8 rats per group): a control group (no surgery), a positive control group (no AIHP treatment before surgery), and three experimental groups (AIHP treatment before surgery). The experimental groups received 207.5, 415, or 830 mg/kg AIHP daily by oral gavage for 28 days. On day 28, the rats were fasted, and on day 29, they were anesthetized with 2% isoflurane. During surgery, a small midline incision was made below the xiphoid process to expose the abdominal cavity. The pyloric region of the stomach was gently exteriorized and ligated with surgical sutures, taking care to minimize traction and preserve the pyloric blood supply. After ligation, the stomach was repositioned in the abdominal cavity, and the incision was closed with interrupted sutures. Four hours later, the animals were re-anesthetized and euthanized by isoflurane. The stomach was removed, and gastric fluid was collected for analysis of volume and total acidity (pH). The stomach was then opened along the greater curvature. Ulcerative lesions were examined under a magnifying glass, and ulcer diameters were measured with a vernier caliper to calculate the gastric ulcer index. AIHP was administered orally once daily for 28 consecutive days prior to pyloric ligation to assess its preventive effects against subsequent gastric injury. Following pyloric ligation surgery, animals were closely monitored during the 4-hour postoperative period for signs of pain or distress, such as changes in behavior, posture, mobility, respiration, or responsiveness. Humane endpoints were predefined, and any animal exhibiting unexpected severe distress would have been immediately euthanized; however, no animals met these criteria during the study. Given the acute nature of the experimental model and the short observation period, analgesics were not administered, in accordance with the IACUC-approved protocol, to avoid potential confounding effects on gastric secretion and inflammatory parameters.

### Gastric juice collection and acid-base titration

During autopsy, the rat duodenum and esophagus were transected, and the stomach tissue was excised. After harvesting the gastric tissue, it was opened down the greater curvature to collect gastric juice. The volume of gastric juice was recorded. Next, total acidity was measured by performing acid-base titration using 0.1 N NaOH.

### Gastric ulcer index

Assessment of the gastric ulcer index was based on established criteria 31,32: Ulcer index = 0: normal stomach; Ulcer index = 0.5: red coloration; Ulcer index = 1.0: spot ulcer; Ulcer index = 1.5: hemorrhagic streak; Ulcer index = 2.0: ulcer; Ulcer index = 3.0: perforation. The protective effect was calculated using the following formula:

Protection rate (%) = (ulcer index of pyloric ligation group - ulcer index of treatment group) / ulcer index of pyloric ligation group × 100%

### Immunohistochemical analysis

Immunohistochemical staining was performed as follows. Tissue sections were deparaffinized and rehydrated before incubation in 3% hydrogen peroxide solution for 30 minutes to block endogenous peroxidase activity. Antigen retrieval was performed by boiling the sections in 0.01 M citrate buffer for 20 minutes, followed by washing in Tris-HCl buffer containing 0.05% Tween-20 for 3 minutes. Sections were then blocked with blocking buffer for 30 minutes at room temperature. Subsequently, the sections were incubated for 1 hour with primary antibodies against TNF-α (1:200; GeneTex, Hsinchu, Taiwan), IL-1β (1:200; GeneTex), NF-κB (1:200; Proteintech, New Taipei City, Taiwan), or p38 MAPK (1:200; GeneTex). After washing, appropriate secondary antibodies were applied, followed by incubation with a peroxidase-labeled streptavidin-biotin complex. Immunoreactivity was visualized using diaminobenzidine (DAB) substrate. Semi-quantitative evaluation of cytosolic staining was conducted by assessing 50 cells per sample, scoring staining intensity as follows: no staining as 1, weak staining as 2, moderate staining as 3, and strong staining as 4. The average immunoreactivity score was calculated by multiplying the number of cells at each staining intensity level by the corresponding coefficient (1-4), summing these products, and dividing by the total number of cells analyzed.

### Cell viability assay

RGM1 cells were obtained from Riken Cell Bank (Tsukuba, Japan). After thawing, the cells were cultured in Dulbecco's Modified Eagle Medium/Ham's F-12 Nutrient Mixture supplemented with 10% fetal bovine serum. An MTT assay was then performed to evaluate the protective effect of acteoside, the major functional component of AIHP, on RGM1 cells. The results showed a reduction in cell viability when acteoside doses exceeded 1 μg/ml. Therefore, the following acteoside concentrations were used for pretreatment in the five experimental groups: 0.1, 0.25, 0.5, 0.75, and 1 μg/ml. Including the control and positive control groups, a total of seven groups were assessed in the MTT assay. RGM1 cells were seeded in 96-well plates (density: 1 × 10^5 viable cells/mL; 100 μL/well) and cultured overnight until adherence. After 24 h of acteoside pretreatment, the culture media in the positive control and experimental groups were replaced with 0.5 mM indomethacin, followed by a 4-hour incubation.

### shRNA targeting p38 MAPK

RGM1 cells were seeded at 1 x 10^6^ cells per well in six-well culture plates and incubated until reaching 50-70% confluence. Cells were then transfected with lentiviral expression system for p38MAPK shRNA (provided by the National RNAi Core Facility, Academia Sinica, Taiwan) using Xfect Transfection Reagent (Clontech, Palo Alto, CA, USA), following the manufacturer's instructions. Forty-eight hours after transfection, total protein was isolated from the cells.

### Western blotting

Harvested RGM1 cells were placed on ice and lysed with cold radioimmunoprecipitation assay (RIPA) buffer (Energenesis Biomedical, Taipei, Taiwan) containing a phosphatase inhibitor (MedChemExpress, Monmouth Junction, NJ, USA) and a protease inhibitor (Future Scientific, Taoyuan, Taiwan). The mixture was shaken for 1 hour at 4°C to produce homogenates, which were then transferred to Eppendorf tubes and centrifuged at 13,000 rpm at 4°C. The supernatant was collected from each sample. Each quantified protein sample was mixed with loading buffer and heated at 95°C for 30 minutes. The samples were separated by 10% sodium dodecyl sulfate-polyacrylamide gel electrophoresis (SDS-PAGE). The resulting bands were electrotransferred onto polyvinylidene fluoride (PVDF) membranes. The membranes were blocked with BlockPro buffer (Visual Protein, Taipei, Taiwan), cut according to the molecular weight of the target protein, and incubated with primary and secondary antibodies. Antibodies were diluted in blocking buffer according to the manufacturers' instructions. The following primary antibodies were used for Western blotting: anti-TNF-α (1:1000; GeneTex, Hsinchu, Taiwan), anti-IL-6 (1:1000; GeneTex), anti-IL-1β (1:1000; GeneTex), anti-NF-κB p65 (1:2000; GeneTex), anti-p38 MAPK (1:1000; GeneTex), mouse anti-β-actin (1:10,000; Proteintech, New Taipei City, Taiwan), and anti-GAPDH (1:10,000; Proteintech). The secondary antibodies were horseradish peroxidase-conjugated goat anti-rabbit immunoglobulin G (1:10,000; Jackson ImmunoResearch, West Grove, PA, USA) and anti-mouse immunoglobulin G (1:20,000; arigo, Hsinchu, Taiwan). Protein expression was detected using an enhanced chemiluminescence solution (Biokit Biotechnology Incorporation, Miaoli, Taiwan) on a FUSION Solo S Chemiluminescence Imaging System (VILBER, Seine-et-Marne, Île-de-France, France). Band densitometry was analyzed with ImageJ software.

### Statistical analysis

All data are presented as mean ± standard deviation (SD). Data analysis was performed using GraphPad Prism (version 9, GraphPad Software, San Diego, CA, USA). Groups differences were assessed by one-way analysis of variance (ANOVA), followed by Tukey's post hoc test for multiple comparisons. For comparisons involving only two groups, an unpaired Student's t-test was used. Normality and homogeneity of variances were assessed using the Shapiro-Wilk and Levene's tests, respectively. Differences with a p-value below 0.05 were regarded as statistically significant. In the toxicity study, significant findings were interpreted with reference to historical control ranges and dose-response trends to distinguish between incidental and treatment-related effects.

## Results

### AIHP did not inhibit gastric acid secretion or acidity in the pyloric ligation model

The gastric acid volumes in the control, positive control, 0.5-fold AIHP, 1-fold AIHP, and 2-fold AIHP groups were 0.25 ± 0.16, 2.55 ± 0.91, 4.25 ± 1.74, 3.22 ± 1.29, and 3.59 ± 1.79 mL, respectively. The corresponding pH values were 2.0 ± 0.3, 1.3 ± 0.3, 1.3 ± 0.2, 1.2 ± 0.1, and 1.3 ± 0.2. Notably, there were no significant differences in either gastric acid volume or pH between the positive control and AIHP-treated groups (Table 1).

### AIHP reduced the gastric ulcer index in the pyloric ligation model

**Figure 2:**
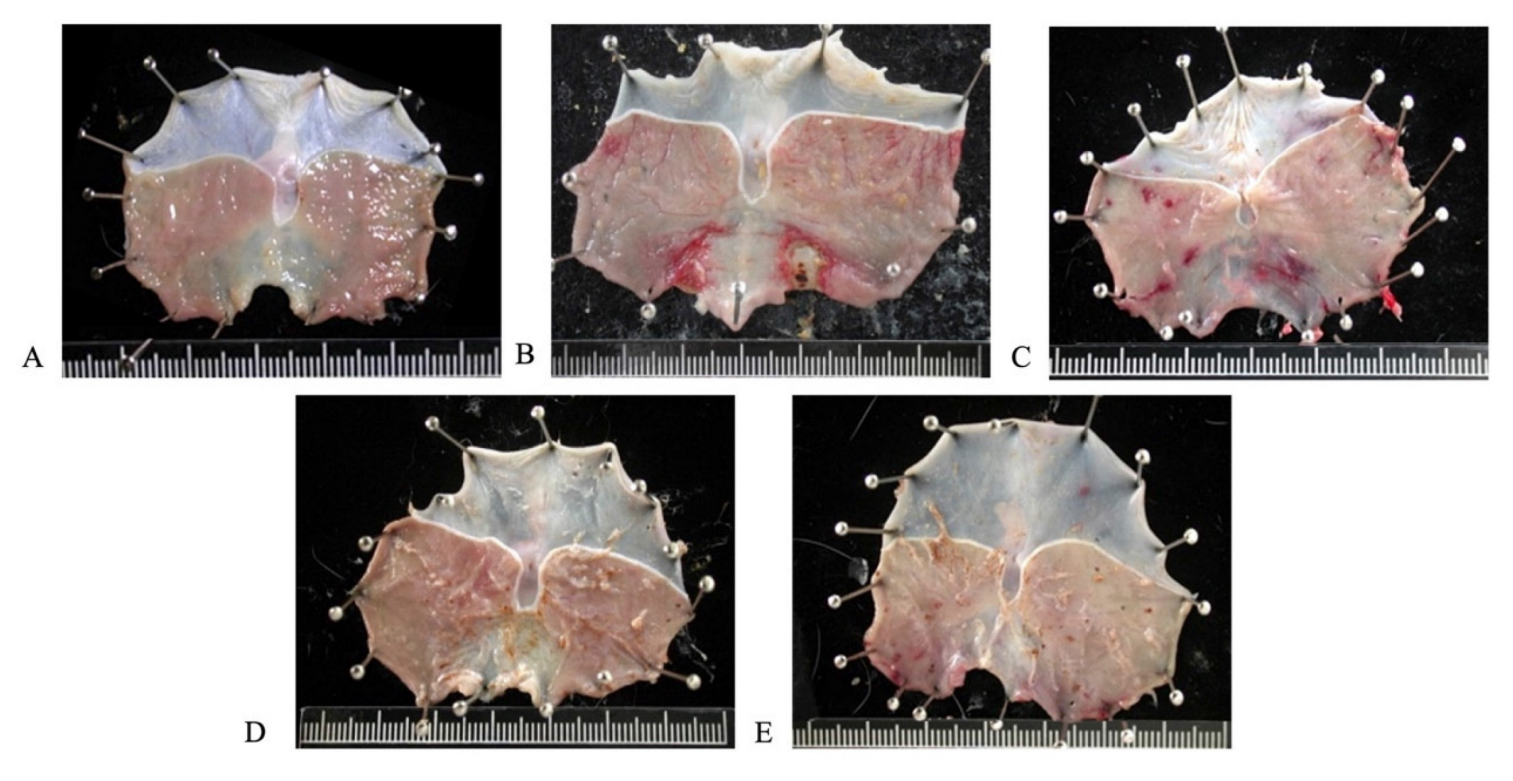
** Gastric mucosa gross finding of AIHP in Wistar rats.** (A) Control group, (B) pyloric ligation control group, (C) 207.5 mg/kg body weight and pyloric ligation group, (D) 415 mg/kg body weight and pyloric ligation group, and (E) 830 mg/kg body weight and pyloric ligation group. (n=8).

Pretreatment with AIHP for 28 days significantly reduced the ulcer index and gastric mucosal damage induced by pyloric ligation. The gastric mucosa remained intact and undamaged in the control group (Figure 2A), while the *pyloric* ligation group (Figure 2B) showed bleeding and ulceration. The gastric ulcer indices for the *pyloric* ligation, 0.5-fold AIHP (Figure 2C), 1-fold AIHP (Figure 2D), and 2-fold AIHP (Figure 2E) groups were 2.06 ± 0.62, 0.69 ± 0.75, 0.31 ± 0.37, and 0.25 ± 0.5, respectively (Table 2). The gastric mucosal protection rates were 66.63% ± 36.55% for 0.5-fold AIHP, 84.83% ± 18.06% for 1-fold AIHP, and 87.86% ± 25.95% for 2-fold AIHP (Table 2).

### AIHP downregulated the expression of TNF-α, IL-1β, NF-κB, and p38 MAPK in the pyloric ligation model

**Figure 3:**
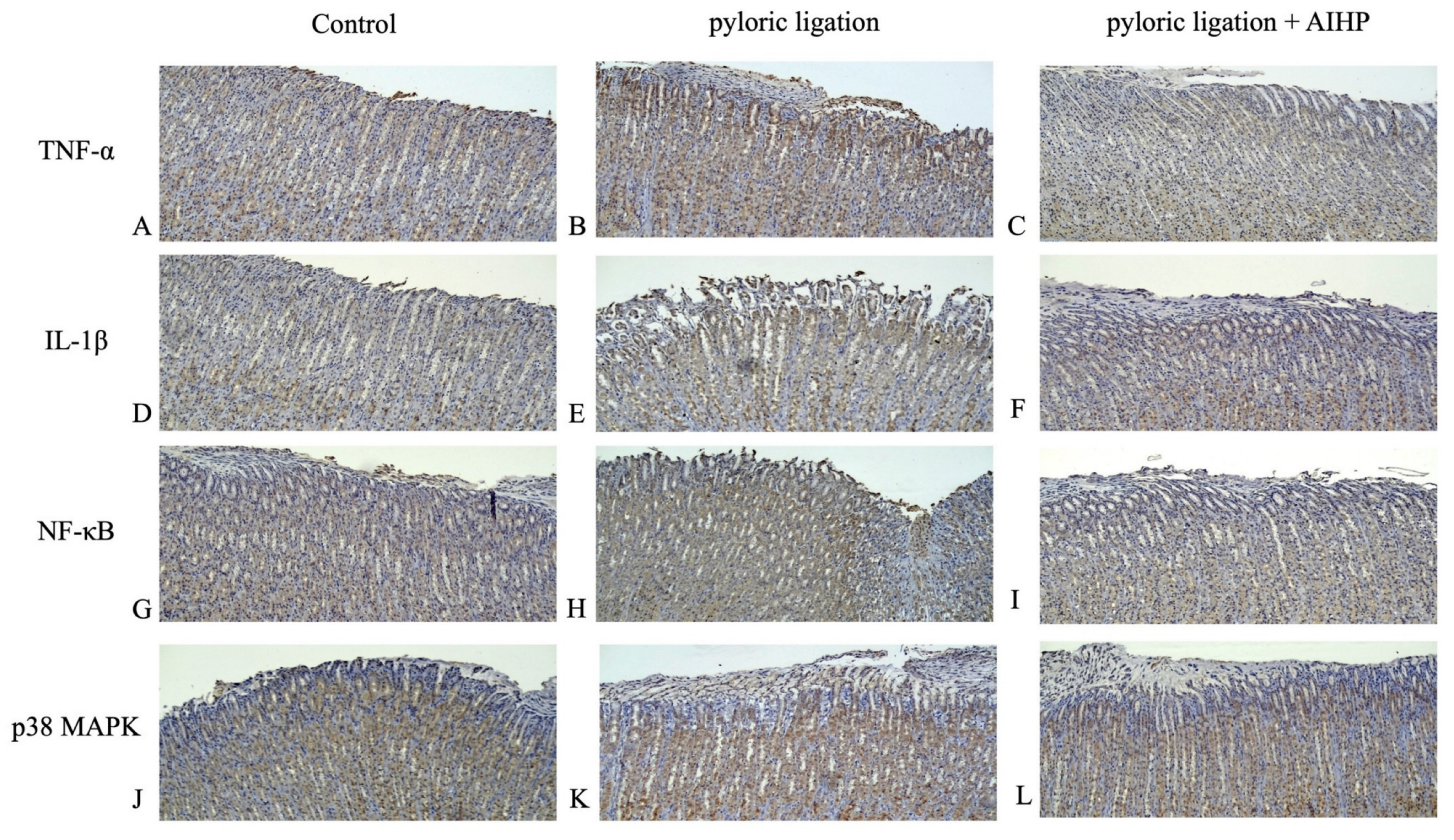
** AIHP decreased pyloric ligation-induced TNF-α , IL-1β, NF-κB, and p38 MAPK expression.** TNF-α protein expression (A) Control group, (B) pyloric ligation control group, (C) AIHP + pyloric ligation group; IL-1β protein expression (D) Control group, (E) pyloric ligation group, (F) AIHP + pyloric ligation group; NFκB protein expression (G) Control group, (H) pyloric ligation control group, (I) AIHP + pyloric ligation group; p38 MAPK protein expression (J) Control group, (K) pyloric ligation control group, (L) AIHP + pyloric ligation group. The brown-stained area is where the protein is expressed. The average intensities of TNF-α expression by semi-quantitative IHC were 1.18 ± 0.06, 2.42 ± 0.01, and 1.52 ± 0.07, respectively (M); IL-1β expression by semi-quantitative IHC were 1.27 ± 0.01, 2.72 ± 0.11, and 1.67 ± 0.13, respectively; NF-κB expression by semi-quantitative IHC were 2.27 ± 0.01, 3.29 ± 0.04, and 2.48 ± 0.02, respectively; and P38 MAPK expression by semi-quantitative IHC were 1.19 ± 0.03, 3.06 ± 0.07, and 2.02 ± 0.09, respectively. Data are expressed as mean ± SD (n = 8). Statistical analysis was performed using one-way ANOVA followed by Tukey's post hoc test. *p* < 0.05 was considered statistically significant.

The protein expression levels of TNF-α in the gastric mucosa of rats in the control group, pyloric ligation group, and AIHP group are shown in Figures 3A, 3B, and 3C, respectively. IL-1β expression levels are shown in Figures 3D, 3E, and 3F; NF-κB expression levels are shown in Figures 3G, 3H, and 3I; and p38 MAPK expression levels are shown in Figures 3J, 3K, and 3L, respectively. Semi-quantitative IHC analysis revealed that the average staining intensities of TNF-α were 1.18 ± 0.06 in the control group, 2.42 ± 0.01 in the pyloric ligation group, and 1.52 ± 0.07 in the AIHP group (Table 3). Similarly, IL-1β expression levels were 1.27 ± 0.01, 2.72 ± 0.11, and 1.67 ± 0.13; NF-κB expression levels were 2.27 ± 0.01, 3.29 ± 0.04, and 2.48 ± 0.02; and p38 MAPK expression levels were 1.19 ± 0.03, 3.06 ± 0.07, and 2.02 ± 0.09, in the control, pyloric ligation, and AIHP groups, respectively.

### Acteoside increased the viability of indomethacin-treated RGM1 cells

**Figure 4:**
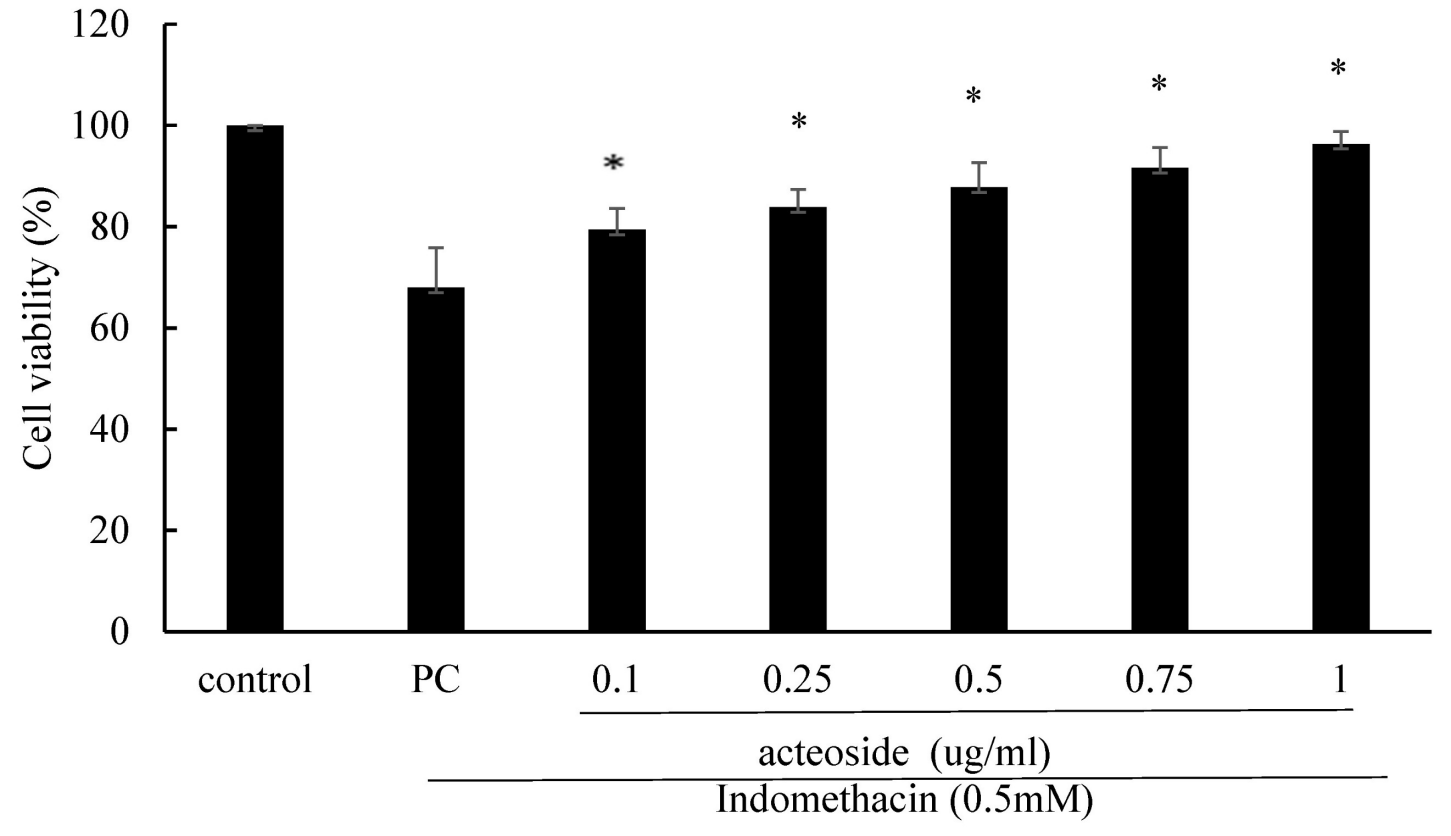
** The effect of acteoside on RGM1 cell viability after 0.5 mM indomethacin treatment.** RGM1 cells were pretreated with acteoside (0.1-5 μg/mL) for 24 h, followed by exposure to 0.5 mM indomethacin for 4 h. Cell viability was assessed using the MTT assay. Data are expressed as mean ± SD (n = 3). *p < 0.05 vs. indomethacin-treated group. Statistical analysis was performed using one-way ANOVA followed by Tukey's post hoc test. *p* < 0.05 was considered statistically significant.

Acteoside pretreatment significantly mitigated the reduction in RGM1 cell viability induced by 0.5 mM indomethacin. The viability of RGM1 cells treated with 0.5 mM indomethacin for 4 hours was 68.0% ± 7.9% (Figure 4). The viability percentages of RGM1 cells pretreated with 0.1, 0.25, 0.5, 0.75, and 1 mg/mL acteoside for 24 hours, followed by 0.5 mM indomethacin treatment for 4 hours, were 79.4% ± 4.2%, 83.9% ± 3.5%, 87.8% ± 4.8%, 91.6% ± 4.1%, and 96.4% ± 2.4%, respectively (Figure 4). Pretreatment with acteoside significantly increased the viability of indomethacin-treated RGM1 cells (*p* < 0.05).

### Acteoside downregulated the expression of TNF-α , IL-1β, IL-6, NF-κB, and p38 MAPK in indomethacin-treated RGM1 cells

**Figure 5:**
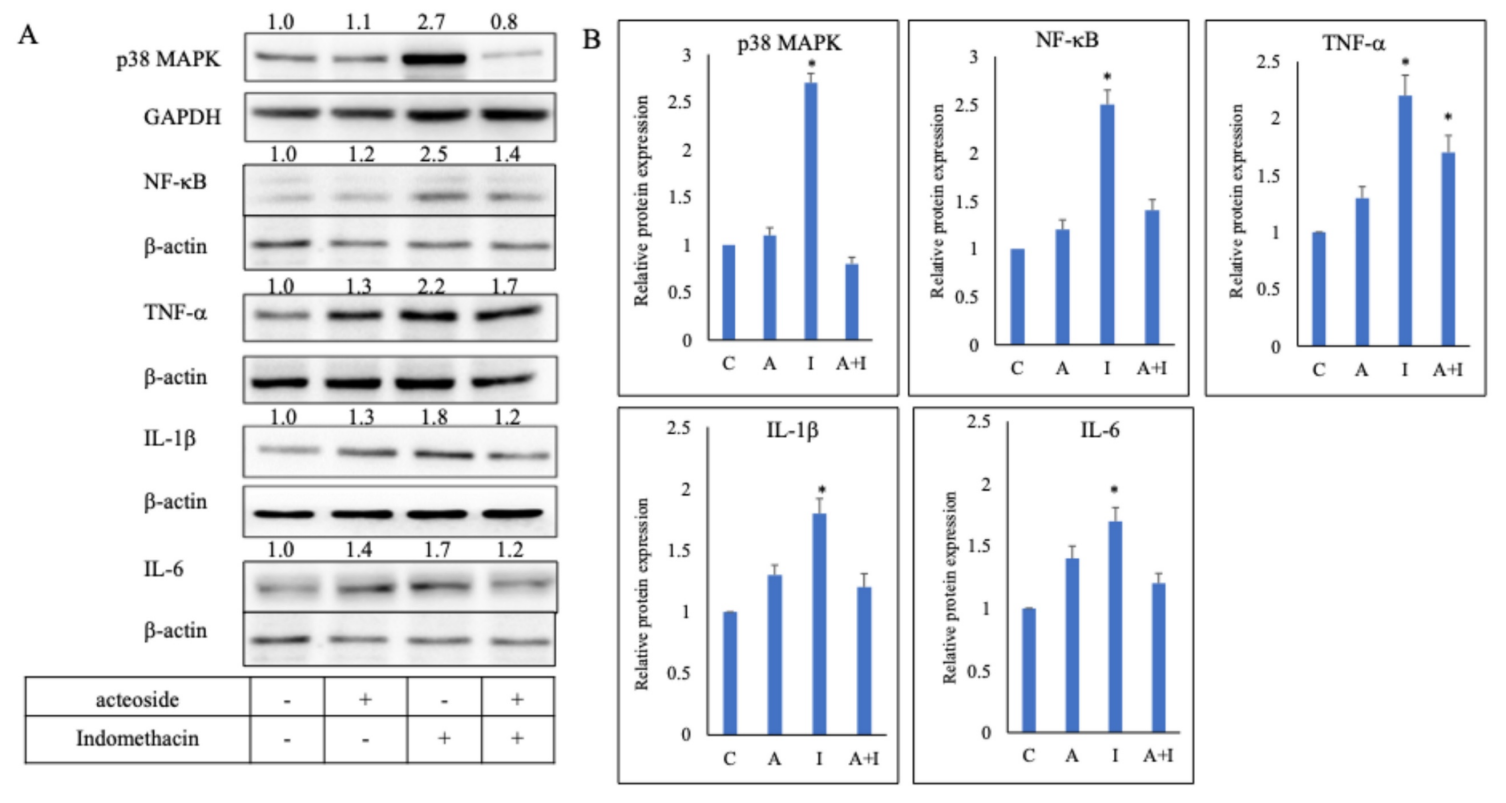
** Effect of acteoside on the inflammation pathway.** RGM1 cells were pre-treated with acteoside for 24 h, and treated with indomethacin 0.5 mM. Acteoside inhibited indomethacin-induced overexpression of p38 MAPK, NF-κB, TNF-α, IL-1β, and IL-6 (A). Indomethacin increased the relative protein expression of p38 MAPK, NF-κB, TNF-α, IL-1β, and IL-6 to 2.7, 2.5, 2.2, 1.8, and 1.7 times that of control (B). Following acteoside treatment, the relative expression levels of these proteins decreased to 0.8, 1.4, 1.7, 1.2, and 1.2, respectively. Data are expressed as mean ± SD (n = 3). Statistical analysis was performed using one-way ANOVA followed by Tukey's post hoc test. *p* < 0.05 was considered statistically significant.

Culture media from RGM1 cells pretreated with 0.1 μg/mL acteoside for 24 h, and from those without pretreatment (positive control), were replaced with 0.5 mM indomethacin and incubated for 4 h. Acteoside pretreatment inhibited indomethacin-induced overexpression of p38 MAPK, NF-κB, TNF-α, IL-1β, and IL-6 (Figure 5A). In the indomethacin group, the relative expression levels of p38 MAPK, NF-κB, TNF-α, IL-1β, and IL-6 were 2.7, 2.5, 2.2, 1.8, and 1.7, respectively. In contrast, in the acteoside + indomethacin group, their relative expression levels were reduced to 0.8, 1.4, 1.7, 1.2, and 1.2, respectively (Figure 5B).

### Acteoside downregulated the expression of TNF-α, IL-1β, IL-6 following p38 MAPK knockdown by shRNA

**Figure 6:**
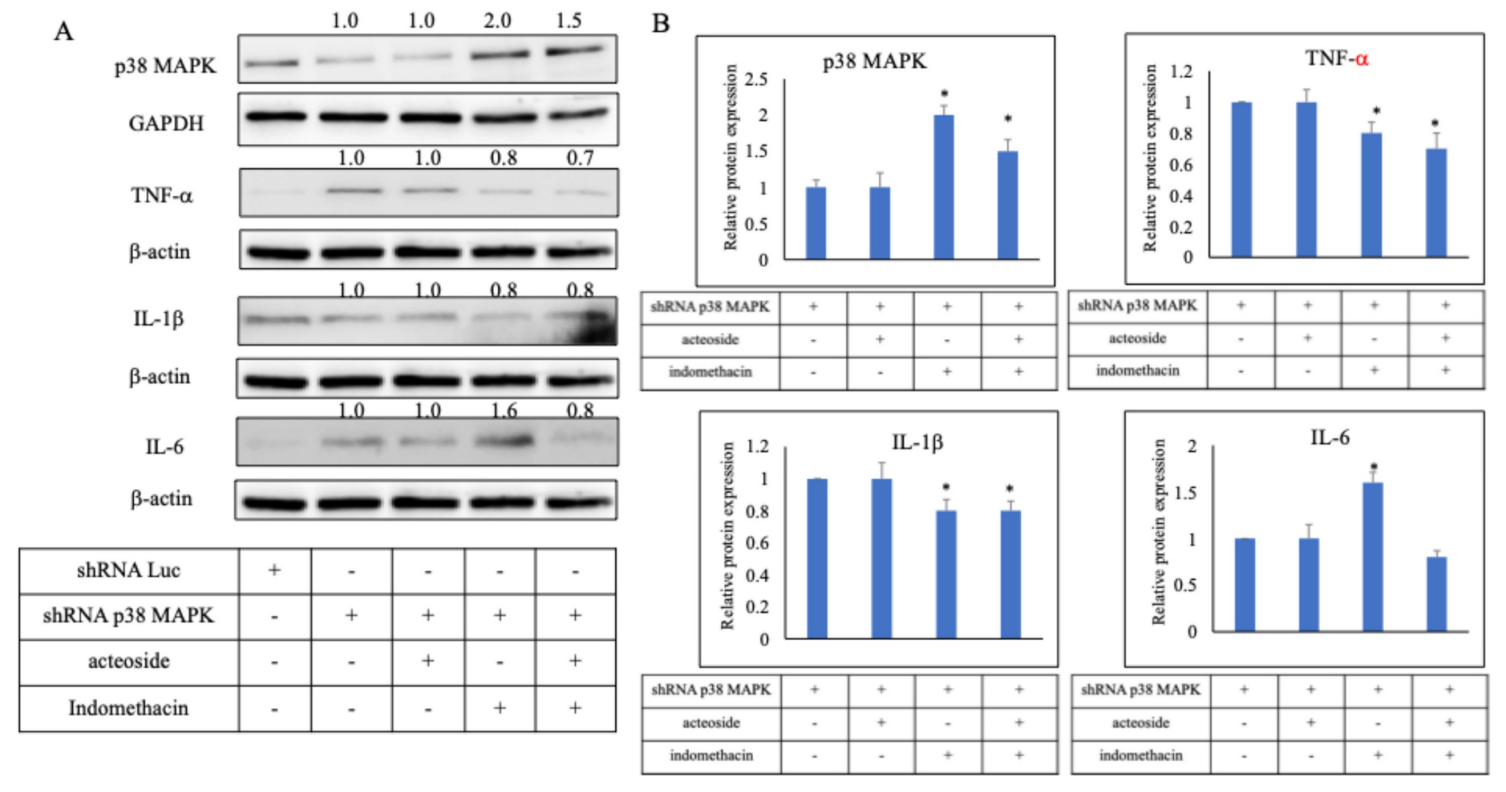
** Effect of acteoside on the inflammation pathway by shRNA p38 MAPK.** p38 MAPK-specific shRNA was used for gene knockdown (A). In the p38 MAPK shRNA-transfected group, protein expression was reduced to 0.65 ± 0.02, confirming effective silencing of the target gene. Relative protein expression levels were then evaluated in the combined treatment groups compared with the p38 MAPK shRNA group. The p38 MAPK protein displayed relative expression levels of 1.0 (shRNA + acteoside), 2.0 (shRNA + indomethacin), and 1.5 (shRNA + acteoside + indomethacin). TNF-α expression levels were 1.0, 0.8, and 0.7, respectively; IL-1β levels were 1.0, 0.8, and 0.8; and IL-6 levels were 1.0, 1.6, and 0.8, respectively (B). Data are expressed as mean ± SD (n = 3). Statistical analysis was performed using one-way ANOVA followed by Tukey's post hoc test. *p* < 0.05 was considered statistically significant.

To further clarify the mechanism of acteoside, p38 MAPK-specific shRNA was used for gene knockdown. In the p38 MAPK shRNA-transfected group, protein expression was reduced to 0.65 ± 0.02, confirming effective silencing of the target gene. Relative protein expression levels were then evaluated in the combined treatment groups compared with the p38 MAPK shRNA group (Figure 6A). The p38 MAPK protein displayed relative expression levels of 1.0 (shRNA + acteoside), 2.0 (shRNA + indomethacin), and 1.5 (shRNA + acteoside + indomethacin). TNF-α expression levels were 1.0, 0.8, and 0.7, respectively; IL-1β levels were 1.0, 0.8, and 0.8; and IL-6 levels were 1.0, 1.6, and 0.8, respectively (Figure 6B). Because p38 MAPK was silenced in all shRNA-transfected groups, the ability of indomethacin to induce TNF-α and IL-1β expression was markedly diminished, resulting in relatively low cytokine levels when normalized to the shRNA + acteoside group.

## Discussion

The present study was designed to investigate the preventive effects of AIHP on gastric ulcer development. Because AIHP was administered prior to ulcer induction, the findings should be interpreted as evidence of preventive gastroprotection rather than therapeutic efficacy against established gastric ulcers. Whether AIHP provides therapeutic benefits after ulcer formation warrants further investigation. We evaluated the gastroprotective effects of AIHP in a pyloric ligation-induced gastric ulcer model in rats. Although AIHP had no significant impact on gastric acid secretion or acidity following pyloric ligation, it markedly reduced the gastric ulcer index. Excessive gastric acid secretion is a well-known contributing factor to gastric ulcer development and is associated with *H. pylori* infection 34. Epidemiological studies indicate that *H. pylori* infection is present in approximately 70% of patients with gastric and duodenal ulcers 10. Sterilization and eradication therapy with specific antibiotics can reduce the rate of peptic ulcer recurrence from 75% to 5% 35. Currently, the primary treatment approach for gastric ulcers is “triple therapy,” which involves a proton pump inhibitor plus two antibiotics. After one week of continuous use, the antibiotics are discontinued while the proton pump inhibitor is continued until the end of treatment 36,37. Inappropriate administration of oral nonsteroidal anti-inflammatory drugs (NSAIDs) is a well-established risk factor for peptic ulcer development and upper gastrointestinal bleeding 38. To prevent drug-drug interactions that reduce treatment efficacy and lead to peptic ulcer recurrence, patients must be instructed to use medications correctly.

Proton pump inhibitors (PPIs) mitigate ulcer formation primarily by irreversibly inhibiting the gastric H⁺/K⁺-ATPase enzyme in parietal cells, resulting in profound suppression of acid production 39. In contrast, AIHP showed no significant acid-reducing effect in this model. Instead, its gastroprotective efficacy is attributed to the inhibition of proinflammatory mediators such as TNF-α, IL-1β, NF-κB, and p38 MAPK. These findings strongly suggest that AIHP protects the gastric mucosa primarily through an acid-independent, anti-inflammatory mechanism, distinguishing it from conventional acid-suppressive therapies 40. Such a mechanism may offer potential benefits for patients who cannot tolerate acid-suppressive therapy or require long-term NSAID use. Additionally, unlike PPIs-which may cause adverse effects such as altered gut microbiota or nutrient malabsorption with prolonged use 41-AIHP represents a promising plant-based alternative with a distinct pharmacological profile.

In the pyloric ligation-induced gastric ulcer model in rats, AIHP markedly decreased the gastric ulcer index and protected the gastric mucosa. As the AIHP dosage increased, the gastric ulcer index progressively declined from 0.69 ± 0.75 in the 0.5-fold AIHP group to 0.25 ± 0.5 in the 2-fold AIHP group. The mucosal protection rates also increased with higher dosages, reaching 87.86% ± 25.95% in the 2-fold AIHP group. These findings indicate that AIHP exerts a dose-dependent gastroprotective effect. The mechanisms underlying this protection may involve alleviation of inflammation, mitigation of gastric acid-induced damage, and promotion of mucosal repair. Interestingly, AIHP pretreatment increased gastric juice volume while simultaneously reducing ulcer indices. Although increased gastric volume is often linked to greater mucosal exposure to luminal contents, gastric injury is not determined solely by acid quantity. Rather, ulcer formation depends on a dynamic balance between aggressive factors and mucosal defense mechanisms. The reduction in ulcer severity observed in AIHP-treated animals suggests that AIHP confers gastroprotection primarily by enhancing mucosal defense and suppressing inflammatory signaling, rather than by inhibiting gastric secretion. Similar dissociations between gastric volume and ulcer severity have been reported in studies where anti-inflammatory or cytoprotective agents preserve mucosal integrity despite unchanged or increased gastric secretion. The Shay ulcer rat model is a reliable tool for studying gastric ulcers, particularly those caused by gastric hyperacidity 29,42. However, the Shay model is limited by its inability to replicate the full pathogenesis of gastric ulcers in humans; surgical trauma and stress responses may influence outcomes 43. The Shay model primarily induces gastric ulcers through pyloric ligation and hyperacidity, which differs from the multifactorial and spontaneous development of ulcers in humans. Human gastric ulcers are attributable to multiple factors, such as NSAID use and *H. pylori* infection, which are not fully represented in the Shay ulcer model 44. The pyloric ligation (Shay) model is a classical and reproducible experimental system that primarily reflects acute, acid-driven gastric mucosal injury associated with gastric hypersecretion and surgical stress. Unlike prevalent clinical gastric ulcer etiologies—such as chronic NSAID use or *H. pylori* infection—this model does not recapitulate the multifactorial and chronic nature of human gastric ulcer disease. Therefore, the findings of the present study should be interpreted within the context of acid-related mucosal injury, and extrapolation to human gastric ulcer pathogenesis should be approached with appropriate caution. Nevertheless, because excessive gastric acid secretion and inflammation remain key contributors to mucosal damage, the pyloric ligation model provides a valuable platform for evaluating gastroprotective mechanisms relevant to acid-induced injury and inflammatory signaling pathways. Only male Wistar rats were used in this study to minimize variability related to hormonal fluctuations. However, this is a limitation, as potential sex-specific differences in gastric ulcer susceptibility and gastroprotective responses were not assessed. Future studies including both sexes will be necessary to determine whether the observed protective effects of AIHP are influenced by biological sex. Although prolonged daily oral gavage may be associated with chronic stress, no gastric ulceration or elevation of inflammatory markers was observed in the control group that received vehicle gavage alone. This suggests that the gavage procedure did not induce overt gastric injury or stress-related inflammation under these experimental conditions. Nevertheless, the potential impact of subtle or systemic stress responses not directly assessed cannot be completely excluded.

In this study, AIHP significantly downregulated the expression of key inflammatory markers, including TNF-α, IL-1β, NF-κB, and p38 MAPK, in the gastric mucosa of rats subjected to pyloric ligation. These markers are crucial mediators of inflammation and ulcer formation 24. Therefore, AIHP likely modulates inflammatory responses to exert its protective effects. The reductions in TNF-α, IL-1β, NF-κB, and p38 MAPK expression suggest that AIHP may inhibit proinflammatory pathways contributing to gastric ulcer pathogenesis. These findings are consistent with the known anti-inflammatory effects of other plant-based compounds, such as curcumin 40, which has been shown to inhibit the expression of TNF-α, IL-1β, and NF-κB in various inflammatory conditions. Excessive gastric acid secretion or reflux can damage the gastric mucosa and activate NF-κB 45. NF-κB activation increases the secretion of multiple proinflammatory factors, thereby amplifying the inflammatory response of the gastric mucosa and ultimately leading to pathological conditions such as gastritis and gastric ulcer 46. Thus, NF-κB is a major driver of the inflammatory response triggered by excessive gastric acid secretion and a key regulator of mucosal self-repair 45. Inhibiting NF-κB activation can, therefore, mitigate damage caused by gastric hyperacidity and promote gastric mucosal repair 47.

Acteoside effectively inhibited the overexpression of key inflammatory markers in indomethacin-treated RGM1 cells, including p38 MAPK, NF-κB, TNF-α, IL-1β, and IL-6. These markers play crucial roles in gastric ulcer pathogenesis and associated inflammatory responses 48. The indomethacin concentration used in vitro was selected based on its widespread use in gastric epithelial cell models to induce reproducible inflammatory injury, and thus serves as a mechanistic tool rather than a representation of physiological exposure. Therefore, acteoside likely exerts its protective effects by alleviating inflammation at the molecular level. The inhibition of the NF-κB pathway suggests that acteoside interferes with key signaling involved in the initiation and progression of inflammation 49,50. The downregulation of TNF-α, IL-1β, and IL-6 expression further supports the anti-inflammatory potential of acteoside. These findings align with those observed for other natural compounds, such as curcumin and licorice 51,52, which have been shown to suppress TNF-α, IL-1β, IL-6, and other inflammatory markers in various gastric conditions. The inhibition of the p38 MAPK pathways reinforces that acteoside modulates critical inflammatory signaling. As a major signaling molecule, p38 MAPK is activated in response to various stress stimuli-such as NSAID use, *H. pylori* infection, alcohol consumption, and oxidative stress - all of which contribute to the development of gastric ulcers 50. This further supports p38 MAPK's role as a central regulator of indomethacin-induced inflammatory signaling. Under p38 MAPK knockdown, even in the presence of indomethacin, pathway activation was largely suppressed, confirming its pivotal role in the inflammatory response. Moreover, in the shRNA + indomethacin + acteoside group, p38 MAPK expression showed a reduction compared to the shRNA + indomethacin group. This finding suggests that acteoside may exert supplementary anti-inflammatory effects through alternative molecular targets or signaling pathways beyond p38 MAPK. These findings indicate that p38 MAPK is required for indomethacin-induced inflammatory cytokine expression and that silencing p38 MAPK diminishes the pro-inflammatory response. The observation that TNF-α and IL-1β levels in the shRNA + indomethacin group were lower than the shRNA + acteoside baseline reflects the loss of p38 MAPK-dependent inflammatory signaling. Without functional p38 MAPK, pro-inflammatory stimuli such as indomethacin cannot effectively activate downstream cytokine production. Thus, these data confirm that p38 MAPK is a critical mediator of indomethacin-induced inflammatory responses, rather than indicating an anti-inflammatory effect of indomethacin itself. The in vitro concentration of acteoside used for mechanistic studies was selected to probe the sensitivity of inflammatory signaling, rather than to mimic in vivo exposure levels following AIHP administration. Given the complexity of absorption, metabolism, and tissue distribution, direct comparisons between in vitro concentrations and in vivo doses are not straightforward. Accordingly, these mechanistic findings should be interpreted as evidence of pathway-specific anti-inflammatory activity, not as a direct reflection of physiological acteoside concentrations achieved in vivo. Through these mechanisms, p38 MAPK promotes the expression of inflammatory mediators, including TNF-α, IL-1β, and IL-6, and participates in DNA damage repair. Due to its critical role in inflammation and cell fate determination, the p38 MAPK pathway is a major target for therapeutic intervention in inflammatory, degenerative, and neoplastic diseases 53.

## Conclusions

The gastroprotective effect of acteoside appears to be mediated primarily through acid-independent, anti-inflammatory mechanisms, specifically involving the suppression of the NF-κB and p38 MAPK pathways. This action promotes mucosal protection even in a hyperacidic gastric environment. In RGM1 cells, acteoside improved cell viability and attenuated indomethacin-induced overexpression of p38 MAPK, NF-κB, TNF-α, IL-1β, and IL-6. Notably, the protective effect of acteoside was diminished after p38 MAPK knockdown, highlighting the importance of this pathway in mediating its action. Therefore, further mechanistic studies and clinical investigations are needed to confirm the translational potential of acteoside for human use.

## Figures and Tables

**Table 1:** Gastric juice volume and total acidity of rats fed with AIHP for 28 days.

Group	Gastric juice secretion (ml)	pH value of gastric juice
Control	0.25 ± 0.16	2.0 ± 0.3
Positive control	2.55 ± 0.91*	1.3 ± 0.3*
0.5-fold AIHP	4.25 ± 1.74*	1.3 ± 0.2*
1-fold AIHP	3.22 ± 1.29*	1.2 ± 0.1*
2-fold AIHP	3.59 ± 1.79*	1.3 ± 0.2*

Control: free water (sterilized); Positive control: pyloric ligation; 0.5-fold: 207.5 mg/kg; 1-fold: 415 mg/kg; 2-fold: 830 mg/kg. Data are expressed as the mean ± SD (n=8). * Significant difference versus control groups at p < 0.05.

**Table 2:** Protective effects of AIHP in *pyloric* ligation Wistar rats.

Group	Ulcer index	Protection ratio (%)
Control	0.00 ±0.00	-
Positive control	2.06 ± 0.62*	0.00 ± 0.00
0.5-fold AIHP	0.69 ± 0.75*^, #^	66.63 ± 36.55^#^
1-fold AIHP	0.31 ± 0.37*^, #^	84.83 ± 18.06^#^
2-fold AIHP	0.25 ± 0.50*^, #^	87.86 ± 25.95^#^

Control: free water (sterilized); Positive control: pyloric ligation; 0.5-fold: 207.5 mg/kg; 1-fold: 415 mg/kg; 2-fold: 830 mg/kg. Ulcer index=0: normal stomach, Ulcer index=0.5: Red coloration, Ulcer index=1.0: Spot ulcer, Ulcer index=1.5: Hemorrhagic streak, Ulcer index=2.0: Ulcer, Ulcer index=3.0: Perforation. Data are expressed as the mean ± SD (n=8). * Significant difference versus control groups at p < 0.05. ^#^ Significant difference versus positive control groups at p < 0.05.

**Table 3:** Semi-quantitative analysis of immunoreactivity for in gastric mucosa.

	Intensity			
	TNF-α	IL-1β	NF-κB	p38 MAPK
Control	1.18 ± 0.06	1.27 ± 0.01	2.27 ± 0.01	1.19 ± 0.03
PL^a^	2.42 ± 0.01*	2.72 ± 0.11*	3.29 ± 0.04*	3.06 ± 0.07*
AIHP + PL^b^	1.52 ± 0.07^#^	1.67 ± 0.13^#^	2.48 ± 0.02^#^	2.02 ± 0.09^#^

^a^ PL: pyloric ligation; ^b^0.5-fold AIHP + PL: 0.5-fold *Anisomeles indica* HP813 powder + pyloric ligation. * Significant difference versus control groups at p< 0.05. ^#^ Significant difference versus positive control groups at p< 0.05.

## Data Availability

The authors confirm that the data supporting the findings of this study are available within the article.
